# Metric-guided regularisation parameter selection for statistical iterative reconstruction in computed tomography

**DOI:** 10.1038/s41598-019-40837-7

**Published:** 2019-04-12

**Authors:** Sebastian Allner, Alex Gustschin, Andreas Fehringer, Peter B. Noël, Franz Pfeiffer

**Affiliations:** 1 Chair of Biomedical Physics and Department of Physics and Munich School of BioEngineering, Technical University of Munich, 85748 Garching, Germany; 2 MITOS GmbH, 85748 Garching, Germany; 3 Department of Diagnostic and Interventional Radiology, Klinikum rechts der Isar, Technical University of Munich, 81675 München, Germany

## Abstract

As iterative reconstruction in Computed Tomography (CT) is an ill-posed problem, additional prior information has to be used to get a physically meaningful result (close to ground truth if available). However, the amount of influence of the regularisation prior is crucial to the outcome of the reconstruction. Therefore, we propose a scheme for tuning the strength of the prior via a certain image metric. In this work, the parameter is tuned for minimal histogram entropy in selected regions of the reconstruction as histogram entropy is a very basic approach to characterise the information content of data. We performed a sweep over different regularisation parameters showing that the histogram entropy is a suitable metric as it is well behaved over a wide range of parameters. The parameter determination is a feedback loop approach we applied to numerically simulated FORBILD phantom data and verified with an experimental measurement of a micro-CT device. The outcome is evaluated visually and quantitatively by means of root mean squared error (RMSE) and structural similarity (SSIM) for the simulation and visually for the measured sample (no ground truth available). The final reconstructed images exhibit noise-suppressed iterative reconstruction. For both datasets, the optimisation is robust where its initial value is concerned. The parameter tuning approach shows that the proposed metric-driven feedback loop is a promising tool for finding a suitable regularisation parameter in statistical iterative reconstruction.

## Introduction

Statistical iterative reconstruction (SIR) continues to be a topic of great interest in the Computed Tomography (CT) community^1–6^. This class of algorithms, as opposed to analytical^7^ and algebraic reconstruction techniques^8,9^, is based on Bayesian statistics and solves an inverse problem for the most probable solution given data and prior information. In the context of CT, this usually entails a data model based on Lambert-Beer’s law and prior knowledge in the form of a regularisation term. The described likelihood functions can be solved using various algorithms, e.g. non-linear conjugate gradient (NLCG)^10,11^ or separable paraboloid surrogates (SPS)^12,13^. As these tomographic problems are very ill-posed, prior knowledge becomes crucial for the image quality of the final reconstruction. Although SIR has already been investigated in detail for different applications, the parameter selection for the strength of the regularisation term is still an open problem. In several publications, this parameter is selected by visual inspection using a highly inefficient trial-and-error strategy or using extensive variable sweeps covering a vast range of parameters to find the desired image appearance^14,15^.

There are several methods for selecting the balance of data and regularisation term. The L-curve method sets data and regularisation residuals in relation to each other and tries to find an optimum, at which the change of the parameter has the most influence on the image appearance^16^. Another approach uses path seeking to determine the best parameter from estimating intermediate images between two initial reconstructions with different parameter strengths^17^. Berger *et al*. have presented a practical technique which tunes the regularisation strength in relation to certain image noise in a reference image and uses a feedback loop to achieve a desired image^18^.

Another approach for prior-image-based reconstruction is based on estimating the influence of the prior image from the closed-form solution of the reconstruction problem^19^.

Here, we introduce an automated parameter tuning approach build on tracking an image quality metric in the reconstruction volume and optimising the regularisation parameter accordingly. For the proposed method, a fully converged reconstruction to evaluate the image metric is not necessary. An intermediate trend is sufficient for quality evaluations to compare the result of several regularisation parameters with respect to the metric performance. A stabilisation period defines how many iterations are needed to achieve a reliable result. As an image quality metric, we employ histogram entropy^20^ as it is a fundamental measure for the average amount of information content within a given dataset. We track the histogram entropy for many regularisation parameters revealing a local minimum of the regularisation strength within a predefined parameter range. This makes the histogram entropy a suitable metric for the investigated tomographic problems.

A feedback loop adjusts the regularisation strength in which three similar initial reconstructions are performed starting with three regularisation parameters. The histogram entropy is evaluated for one initial (central), one lower, and one higher parameter, deciding for the ‘best’ regularisation parameter after a certain number of iterations (one optimisation step). The next repetition is started with the best parameter and its reconstruction from the first step. This is repeated until a minimum histogram entropy is found.

First, we apply this method to a numerical experiment with a FORBILD head phantom^21^. For the simulation, a quality check can be performed as the reference image is available. An extensive parameter sweep is performed to compare the best parameter in terms of histogram entropy with the root mean squared error (RMSE) and structural similarity (SSIM). It is also used to evaluate the result of the parameter determination. Secondly, the method is evaluated with experimentally acquired data from a micro-CT device. For both reconstruction cases, the parameter optimisation process is started from several initial parameters selected from a reasonable range to ensure that the same final parameter is found from each starting point.

## Methods

### Statistical Iterative Reconstruction

The iterative reconstruction is performed on the basis of a Gaussian noise model derived from the Bayesian statistic. It assumes Lambert-Beer’s law in the form of a weighted least squares approach. The data term reads:

1 $$D=\sum _{i}\,\frac{{t}_{i}}{2}{({\bar{y}}_{i}-{y}_{i})}^{2},\,\,{\rm{w}}{\rm{i}}{\rm{t}}{\rm{h}}\,{y}_{i}=-\,\mathrm{log}(\frac{{t}_{i}}{{f}_{i}}),\,\,{\rm{a}}{\rm{n}}{\rm{d}}\,{\bar{y}}_{i}=\sum _{j}\,{a}_{ij}{\mu }_{j},$$

where ${t}_{i}$ are the transmission measurements, ${f}_{i}$ the flatfields, and ${y}_{i}$ and ${\overline{y}}_{i}$ are the line integrals and their forward model from the reconstructed linear attenuation coefficients ${\mu }_{j}$. The ${a}_{{ij}}$ represent the system matrix. The projection operations are conducted with a highly efficient projector implementation^22,23^. The reconstruction problem is constrained with a Huber regularisation prior^24^. It penalises differences of neighboring voxels in the reconstruction volume either linearly or quadratically depending on a threshold. Value differences below this threshold are seen as noise and are punished quadratically, and differences above are only treated linearly as they are considered as edges:

2 $${R}_{{\rm{H}}}(\mu ,\gamma )=\sum _{j}\,\sum _{k\in {{\mathscr{N}}}_{j}}\,\{\begin{array}{cc}\frac{{({\mu }_{j}-{\mu }_{k})}^{2}}{2\gamma } & \,{\rm{f}}{\rm{o}}{\rm{r}}\,|{\mu }_{j}-{\mu }_{k}|\le \gamma ,\\ |{\mu }_{j}-{\mu }_{k}|-\gamma /2 & \,{\rm{f}}{\rm{o}}{\rm{r}}\,|{\mu }_{j}-{\mu }_{k}| > \gamma .\end{array}$$

**Figure 1:**
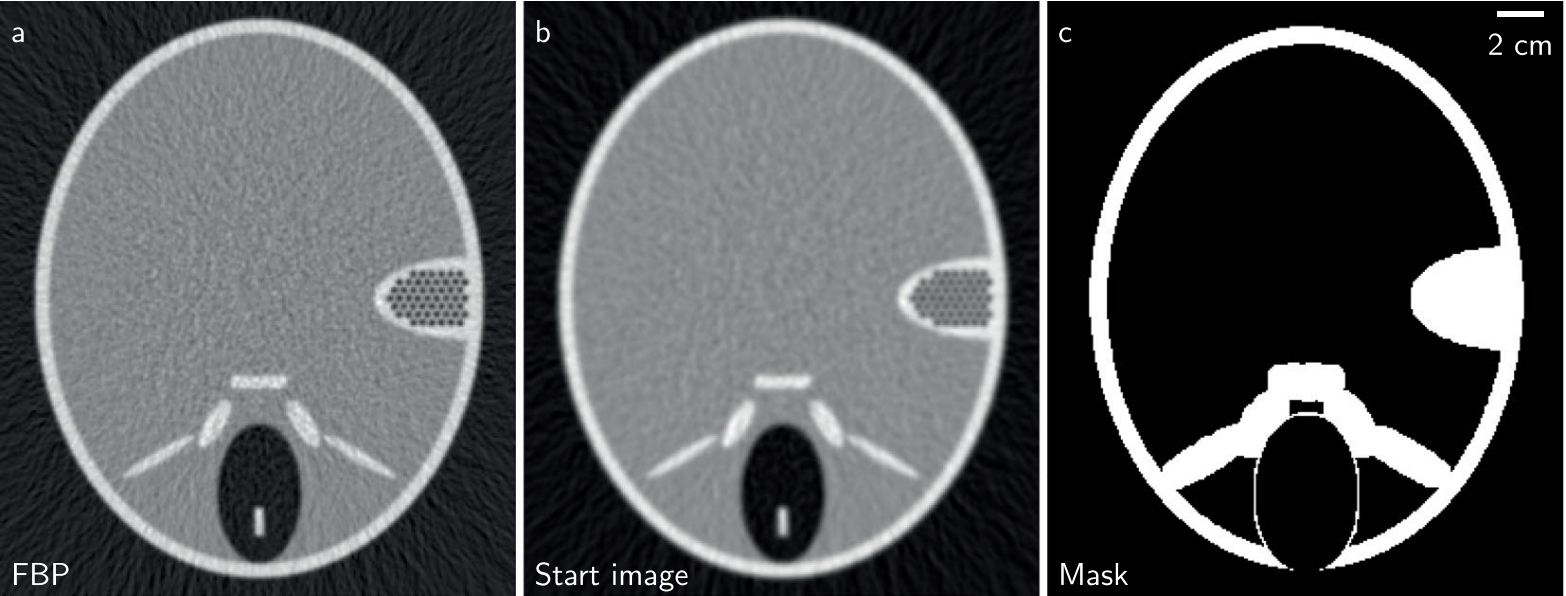
(**a**) Filtered backprojection (FBP); (**b**) Start image for the iterative reconstruction (blurred version of FBP); (**c**) Mask showing which voxels contribute to the histogram entropy. All images show the central slice of the respective volume.

Here, $\mu$ corresponds to different attenuation coefficients in the reconstruction volume with ${\mu }_{k}$ being the central voxel in the neighborhood ${N}_{j}$. As mentioned earlier, the threshold is $\gamma$. A separable paraboloid surrogate solver with momentum transfer (OGM)^13^ was used to accelerate the reconstructions. As a starting image, a blurred version of a FBP reconstruction is used (see Fig. 1). The complete log-likelihood cost function is given by

3 $${\mathscr{L}}(\mu )=\sum _{i}\,\frac{{t}_{i}}{2}{({\bar{y}}_{i}-{y}_{i})}^{2}+\lambda {R}_{{\rm{H}}}(\mu ,\gamma ),\,\,{\rm{w}}{\rm{i}}{\rm{t}}{\rm{h}}\,\hat{\mu }=\mathop{{\rm{a}}{\rm{r}}{\rm{g}}{\rm{m}}{\rm{i}}{\rm{n}}}\limits_{\mu }\,{\mathscr{L}}(\mu ),\,\,{\rm{a}}{\rm{n}}{\rm{d}}\,\lambda  > 0.$$

The parameter $\lambda$ controls the balance between data and regularisation term. In addition, the data and regularization term could be normalised, e.g. by the number of detector pixels, number of image voxels, number of projection angles, or other influence factors to get a more consistent impact of the parameter on differently scaled problems. However, as there is no obvious way for normalisation, the cost function is used as stated in Eq. (3). In analogy to conventional reconstruction with post-processing filters, the parameter $\lambda$ would correspond to the strength of the applied image filter.

### Histogram Entropy

In this work, the metric to evaluate the image quality of the reconstructed image is the histogram entropy^20^ (HE). It grades the content of the image/volume according to basic information in the context of the histogram value distribution. It neglects the spatial distribution of values. The histogram entropy is defined as

4 $$H=-\,{\int }_{{\mu }_{{\rm{\min }}}}^{{\mu }_{{\rm{\max }}}}\,p(\mu )\,\mathrm{log}\,[p(\mu )]\,{\rm{d}}\mu ,$$

with $p(\mu )$ being the continuous distribution function of the gray values. In discrete voxel-based image reconstruction, also the entropy must be calculated discretely. Therefore, we use a pseudo-continuous distribution function with a boxcar function as a Parzen window^25^. It can be regarded as a histogram with non-fixed bin sizes or a convolution of a delta peak distribution of gray values and a boxlike window function of height $\tfrac{1}{hN}$ and width *h*:

5 $$H=-\,\sum _{l=1}^{2N-1}\,{\rm{\Delta }}{g}_{l}\,\frac{{n}_{l}}{hN}\,\mathrm{log}(\frac{{n}_{l}}{N}).$$

In this notation, $l$ defines a section of constant height in the histogram, ${\rm{\Delta }}{g}_{l}$ is the width and ${n}_{l}$ the height of (or the number of contributing voxels to) this section. The total number of considered voxels is $N$. As the entropy is maximised for white noise (see Eq. (4)) the entropy is normalised by ${H}_{{\rm{\max }}}=\,\mathrm{log}(N)$. The selection of the parameter $h$ is not crucial as it only changes the absolute values of the entropy. The qualitative behaviour and relative difference of entropy during reconstruction and parameter selection is very similar. The evaluated histogram entropy is smooth and well-behaved over an extensive range of regularisation parameters, meaning it has a distinct minimum and no additional local minima. A qualitative interpretation of why the histogram entropy can be used to measure image quality is that noise and blur both influence the image histograms. Interpreting Eq. (4), the entropy is maximised for homogeneously distributed noise and minimised for sharp delta peaks. Image noise and blur both widen the histogram peaks which increases the entropy.

### Mask Creation

The histogram entropy is not evaluated over the whole image as to do so would be computationally expensive. Instead, we use a quasi-continuous histogram requiring all contributing values to be sorted. Furthermore, depending on the reconstructed sample, there could be an imbalance of flat regions and edge regions in the image. The influence of large flat regions, such as phantoms, would outweigh the limited impact of fewer blurred edges in a volume. Therefore, we use a mask to decide which voxels contribute to the image metric.

The image metric should favour a decrease in noise while maintaining edges. In addition, the entropy metric only evaluates the gray value histogram, while neglecting the spatial distribution. Therefore, the mask must be calculated from regions containing distinguishable attenuation coefficients in the histogram. To balance these effects, the histogram entropy is evaluated only near edge regions of the image. There, decreasing noise on both sides of the feature edge is favoured by the histogram entropy as long as the edge is maintained. However, if the edge is deteriorated it has a negative influence on the entropy metric.

To obtain the image mask, an edge image is created with an image gradient (central difference) of a blurred version of a FBP reconstruction in three spatial dimensions. Simple thresholding extracts the edges which are then enlarged with a binary dilation operation. Subsequently, the background is excluded by a simple thresholding operation as the background value distribution does not contain relevant information for the sample. The histogram entropy is calculated from the mask content of several slices. The number of slices depends on the mask coverage and sample but should be chosen to produce reliable results while not taking to much time. The mask created in the previously described fashion is shown in Fig. 1.

### Metric-guided Parameter Tuning

The regularisation parameter has a significant influence on the image appearance, and the reconstruction result can be evaluated with an image metric. If this metric has a (local) optimum, the parameter can be tuned accordingly. However, changing the influence of parameter $\lambda$ during one reconstruction does not continue but rather simply restarts the tomographic reconstruction with another initial reconstruction guess (a result of the first SIR) and the new parameter. The relation of data and regularisation term from before the change is out of balance and has a somewhat delayed impact on image formation. During the next iterations, a new balance is determined. The data and regularisation term acts differently on the image, resulting in a possibly non-monotonic change to the new level (with respect to the metric). Therefore, a change of the regularisation strength after each iteration does not lead to the right result because the new image appearance must first stabilise after parameter adaptation (see Supplementary Material for more detail).

In addition, the metric (histogram entropy) does not only change for an adapted parameter but also in the process of continued iterations. Therefore, we utilise a three-parameter approach with three reconstructions to robustly tune the regularisation strength. Three $\lambda$’s are chosen, one with an initial (central), one with a weaker and one with a stronger regularisation with a common starting image (a blurred FBP). From the three reconstructions, the metric identifies the ‘best’ image, and its parameter and intermediate reconstruction are used as the central regularisation strength and as the initial image guess for the next repetition. The parameter update is performed multiplicatively to adapt quickly over orders of magnitude. If the central parameter already yields a better image than the weaker and stronger regularised reconstructions, the best parameter is closer to the central strength. Therefore, the surrounding parameters are refined and set closer to the central one. In the early stages, the stronger and weaker parameter differ quite significantly and become closer to the central parameter as the reconstruction approaches the optimal image in terms of the applied metric. In this paper we use

6 $${\lambda }_{{\rm{stronger}}}^{n}=1+{0.5}^{n}\cdot {\lambda }_{{\rm{central}}},$$

7 $${\lambda }_{{\rm{weaker}}}^{n}={(1+{0.5}^{n})}^{-1}\cdot {\lambda }_{{\rm{central}}},$$

as the multiplicative parameter variation with $n\in {\mathbb{N}}$ being increased if the central parameter suits the metric better than both surrounding regularisation strengths. At the start of the optimisation loop, the weaker regularisation is half as strong, and the stronger regularisation twice as strong as the starting value.

Another way of viewing this approach is as three-point numerical sampling of the possible histogram entropy function in dependent on the iterations and regularisation parameter. It would also be possible to form a numerical gradient (two-point sampling) of this function. However, this is not done here to avoid problems with parameter step size determination. The main advantage of the parameter adaption is that the reconstruction is already relatively close to the current image guess. Therefore, fewer iterations are needed to converge than recommencing from the start image.

### Quantitative Evaluations

To verify the result of the parameter tuning approach based on histogram entropy, we use standard reference-guided quality metrics. The root mean squared error (RMSE) shows the deviation from the ground truth in a straightforward fashion:

8 $${\rm{RMSE}}=\sqrt{\frac{1}{N}\,\sum _{j}\,{w}_{j}{({\mu }_{j}-{\mu }_{j}^{{\rm{ref}}})}^{2}}.$$

According to the equation, a small RMSE corresponds to a reconstruction close to the ground truth. The quantitative errors provided in this work are the relative errors relating to the largest attenuation coefficient in the image.

The structural similarity index (SSIM) is defined to represent human perception in relation to luminance, contrast, and structure^26^. It is calculated using the local mean and standard deviation of the evaluated reconstruction and the reference.

9 $${\rm{SSIM}}=\frac{1}{N}\,\sum _{j}\,{w}_{j}\,{l}_{j}(\overline{\mu },{\overline{\mu }}^{{\rm{ref}}})\,{c}_{j}(\sigma ,{\sigma }^{{\rm{ref}}})\,{s}_{j}(\sigma ,{\sigma }^{{\rm{ref}}},{\sigma }^{{\rm{cc}}}).$$

$\bar{\mu }^\circ$ represents the local mean attenuation coefficients. The $\sigma$° correspond to the local standard deviation of the respective image, and the $\sigma$^cc^ is the local noise covariance of image and reference.

Both metrics were evaluated in the same areas of the sample, which were used to calculate the histogram entropy (see section 2.3). The normalisation $N$ is the number of contributing elements because the weights ${w}_{j}$ are binary (inside or outside the mask).

As these metrics both require a ground truth, they can only be calculated for the simulated FORBILD phantom data.

### Simulation and Scan Parameters

For the numerical experiment, the well known FORBILD head phantom^21^ is used. We performed the simulation at an energy of 70 keV which corresponds to the approximate mean energy of a human head scan for which the values in the FORBILD phantom were designed. The assumed geometry is a parallel beam setup with a 301 px wide detector and 473 projection according to the Nyquist sampling theorem^27^. The binary phantom was created with a four times higher sampling rate and binned afterwards by the same factor. This avoids artificial binary edges and introduces partial volume effects in the discrete phantom sampling. The phantom was simulated with a homogeneous illumination of 5000 photons per pixel for every projection and forward projected with the corresponding linear attenuation coefficients from the NIST-database^28^.

For the experimental evaluation, we used a phantom sample consisting of tubes filled with different concentrations of iodine solution. The concentrations were chosen to have distinct attenuation coefficients in the reconstruction, which is helpful for the applied entropy metric. The measurement was performed with a ZEISS Xradia 500 Versa device at 60 kVp. The sample was scanned with 1000 projections to also fulfill the Nyquist sampling criterion for a detector width of 512 pixels. The pixel size is 135 μm. The distances between source and sample, and between the sample and detector were 5 cm and 8 cm, respectively.

The simulation study and the experimental measurements do not have the same scale with respect to data rays and number of projection angles, because we want to show that the optimum regularisation parameter concerning the image metric can be found for arbitrary reconstruction sizes.

## Results

### Numerical Study

**Figure 2:**
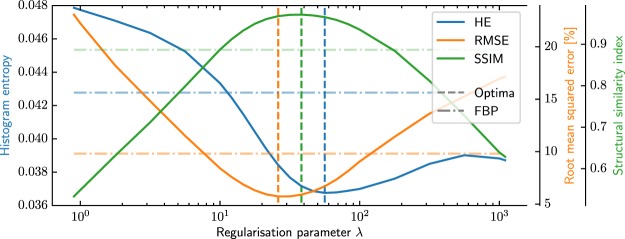
The histogram entropy (HE), root mean squared error (RMSE), and structural similarity index (SSIM) of the statistical iterative reconstruction evaluated close to image edges after 500 iterations for 23 regularisation parameters. The optima of the three image metrics are relatively close considering the tested parameter range. The dashed lines corresponds to the best parameter of the sweep for each metric. The dotted dashed lines correspond the performance of the FBP in the respective metric.

Initially, a wide range of regularisation parameters is tested for a FORBILD head phantom to show that the histogram entropy is a well-behaved metric. This goes from virtually no regularisation to strong over-regularisation spaced in a logarithmic fashion. The parameters are sampled more densely around the histogram entropy minimum. In our implementation we used *λ* $\in$ [0.9, 1, 1.78, 3.16, 5.62, 10, 12, 15, 18, 22, 26, 32, 38, 46, 56, 68, 83, 100, 178, 316, 562, 1000, 1100]. The applied parameters span over three orders of magnitude from very little to strong over-regularisation for 500 iterations each. The resulting reconstructions are evaluated with histogram entropy, root mean squared error (RMSE) and structural similarity (SSIM) with respect to the ground truth of the FORBILD phantom. This can be seen in Fig. 2.

The parameter sweep reveals a minimum in histogram entropy for the regularisation parameter ${\lambda }_{{\rm{best}}}\approx 56$ among the sampled values mentioned in 2.6. The RMSE and SSIM identify the ‘best’ reconstruction with respect to the ground truth phantom at ${\lambda }_{{\rm{best}}}^{{\rm{RMSE}}}\approx 26$, and ${\lambda }_{{\rm{best}}}^{{\rm{SSIM}}}\approx 38$, respectively. However, the histogram entropy does not need a reference and its optimum is reasonably close. Also, the image appearances do not differ considerably which justifies its use as image metric. For completeness, the metrics are also applied to an FBP reconstruction and displayed in the dotted-dashed line. Here, a sharp Ramlak filter and a smooth Hamming filter were used and their best result with respect to the respective metric is shown.

**Figure 3:**
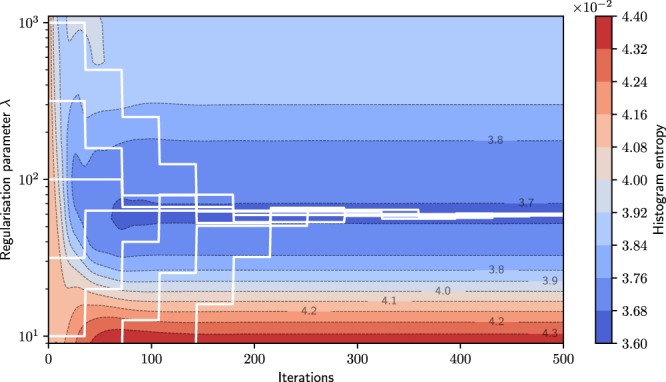
The histogram entropy of the numerical simulation during reconstruction. It is evaluated close to the image edges over 500 iterations for 23 regularisation parameters. The white lines represent the parameter optimisation. The steps show a change of parameter after a stabilisation interval of 35 iterations. The two lines entering the plot from the bottom started at the initial values of ${\lambda }_{{\rm{start}}}\in [1,3]$ outside the displayed range. They approach the final parameter by increasing with ${\lambda }_{{\rm{stronger}}}=2{\lambda }_{{\rm{central}}}$ (see Eq. (6)) over several optimisation steps. During the course of optimisation, the tuning method approaches the minimum for all initial parameters.

The proposed parameter tuning approach is started to find the minimum in histogram entropy identified with the parameter sweep. It should find this local optimum independent of the initial regularisation strength. To prove the independence from its initial value, we optimised the parameter from 7 initial parameters selected from a reasonable range (${\lambda }_{{\rm{init}}}\in [1,3,10,32,100,316,1000]$). The entropy is evaluated within a sweep for all 23 parameters over the 500 iterations represented by the coloured background in Fig. 3 with the contour lines representing different entropy levels. This plot also shows that the reconstructions of the parameter sweep no longer change in respect of entropy after around iteration 150.

**Figure 4:**
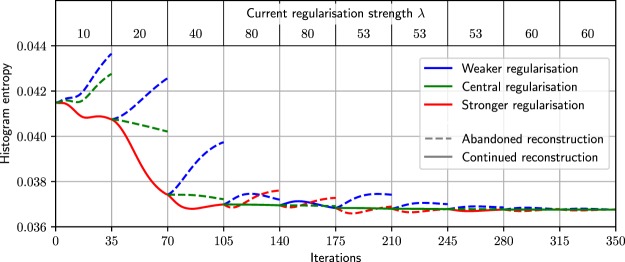
The image entropy during parameter optimisation for the numerical study. The three lines show reduced, central, increased regularisation strength. The dashed lines represent the abandoned reconstruction paths which exhibit a sub-optimal histogram entropy after image stabilisation. This figure shows ten optimisation steps with a total of 350 iterations. During optimisation, the tuning method approaches the minimum entropy. The upper part shows the current regularisation parameter applied during the respective optimisation step. This optimisation starts at ${\lambda }_{{\rm{init}}}=10$ and approaches ${\lambda }_{{\rm{opt}}}=60$.

The white lines in Fig. 3 display the process of optimisation started from the initial positions mentioned before. After a stabilisation period of 35 iterations (one optimisation step), the intermediate reconstructions are evaluated, and the parameter is changed or kept depending on the result of the entropy metric. After five to six optimisation steps, all tuning runs have found a parameter very close to the aforementioned optimum determined with the parameter sweep. The reconstructions are hardly distinguishable after 175 iterations (five optimisation steps) and look the same after ten steps. One tuning run is shown in more detail in Fig. 4 for the example of ${\lambda }_{{\rm{init}}}=10$. It shows the reconstructions histogram entropy changing through iterations and change of regularisation strength.

**Figure 5:**
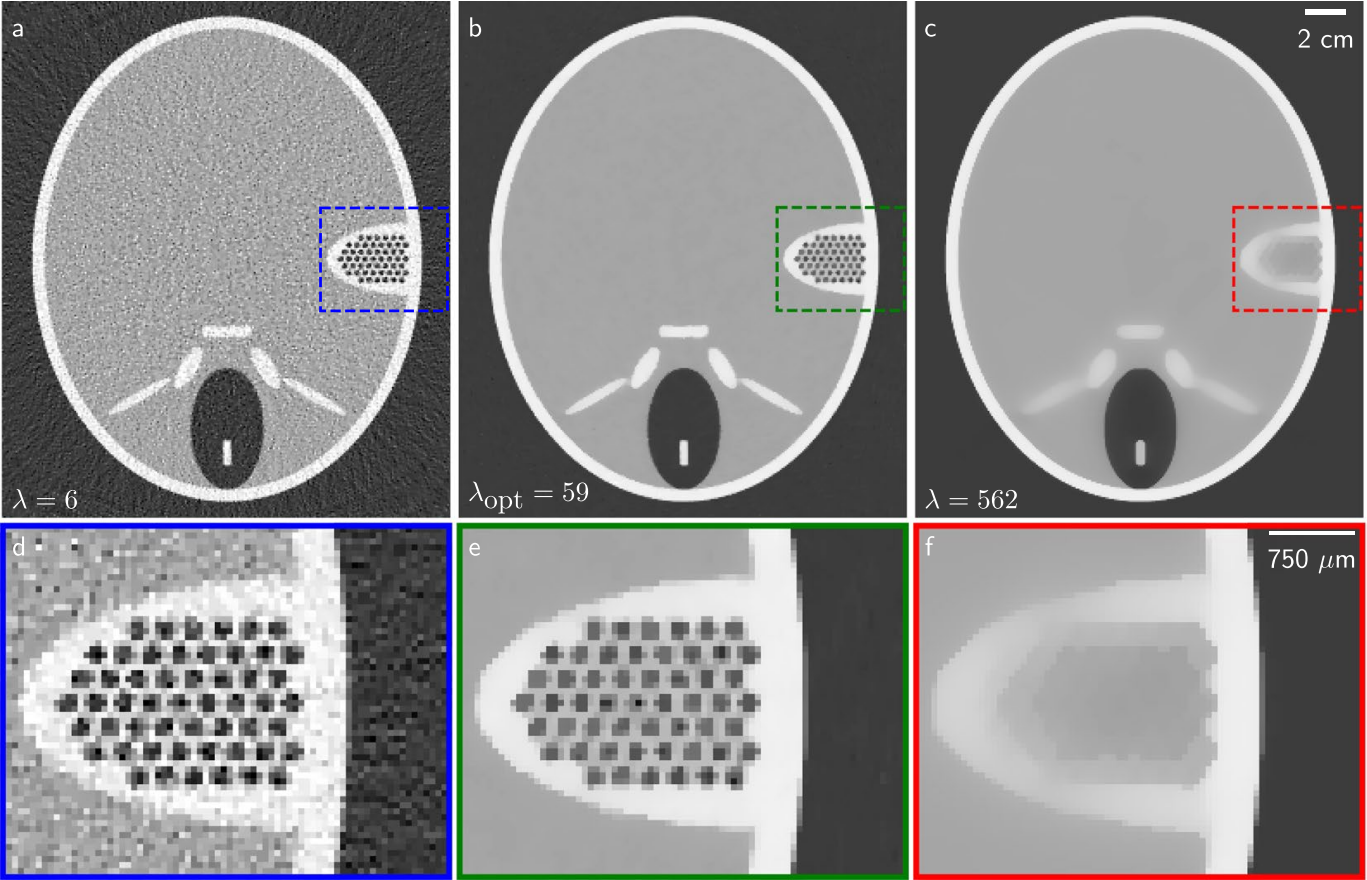
The results of the numerical experiment. (**a**–**c**) The reconstruction from the optimised $\lambda$ parameter is shown in comparison to two regularisation strengths from the parameter sweep. The bottom images (**d**–**f**) show a detail feature of the top row. (**b**) Is the reconstruction with the optimised parameter. Here, noise is suppressed while features in the inner ear of the phantom are still maintained. The parameters are chosen to highlight differences arising from weaker and stronger regularisation. (**a**) Is the under-regularised image without feature loss but with high noise. (**c**) Is an over-regularised image with low noise but feature loss.

For the first optimisation steps, the regularisation parameter is doubled, as the entropy is minimal for the increased regularisation ($\lambda :10\to 20\to 40\to 80$). After increasing the parameter three times, the base or central parameter yields the best result and therefore, the current strength $\lambda =80$ is kept. In the subsequent optimisation step, a smaller parameter span is investigated by adapting the multiplicative factors of Eqs (6) and (7) to be closer to the central strength. When the parameter is adapted, the factors stay the same and when the parameter is kept, the factors are adapted. Finally, the reconstruction result of the optimised regularisation parameter ${\lambda }_{{\rm{opt}}}=59$ is shown in Fig. 5.

Figure 5(a) shows a noisy reconstruction achieved with a reduced regularisation influence. Figure 5(c) displays over-smoothing at the inner ear representation and between the bones around the nose area. This effect increases for even larger $\lambda$ parameters until the head phantom is completely distorted. The reconstruction optimised with respect to image entropy exhibits excellent noise reduction in combination with maintained edges and features. A comparison of the optimised regularisation parameter with FBP reconstructions processed with a smooth Hamming filter and a sharp Ramlak filter can be found in the Supplementary Material.

### Experimental Validation

**Figure 6:**
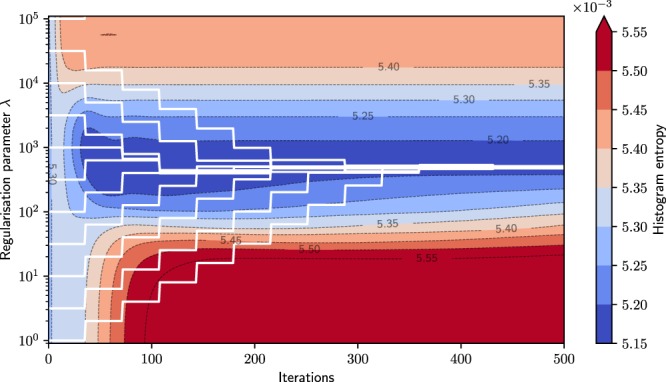
The image entropy for the experimental validation evaluated close to image edges over 500 iterations for 31 regularisation parameters. The white lines represent the parameter optimisation and show a possible change of parameter after the stabilisation intervals of 35 iterations. During the course of optimisation, the tuning method approaches the minimum for all but one initial parameter. For the optimisation run started with the largest initial parameter, the determination fails.

To verify these results with experimental data, we measured different iodine concentrations with distinct uniform attenuation coefficients in plastic tubes. For this measurement, RMSE and SSIM cannot be used because there is no available reference object. A parameter sweep was performed for the regularisation strengths $\lambda \,\in$ [0.9, 1, 2, 3, 6, 10, 18, 32, 56, 100, 121, 147, 178, 215, 261, 316, 383, 464, 562, 681, 825, 1000, 1778, 3162, 5623, 10000, 17783, 31623, 56234, 100000, 110000] (numbers rounded) distributed over five orders of magnitude. Subsequently, the optimisation procedure was performed with starting parameters from across the full sweeping range analogously to the numerical simulation. The results are shown in Fig. 6.

**Figure 7:**
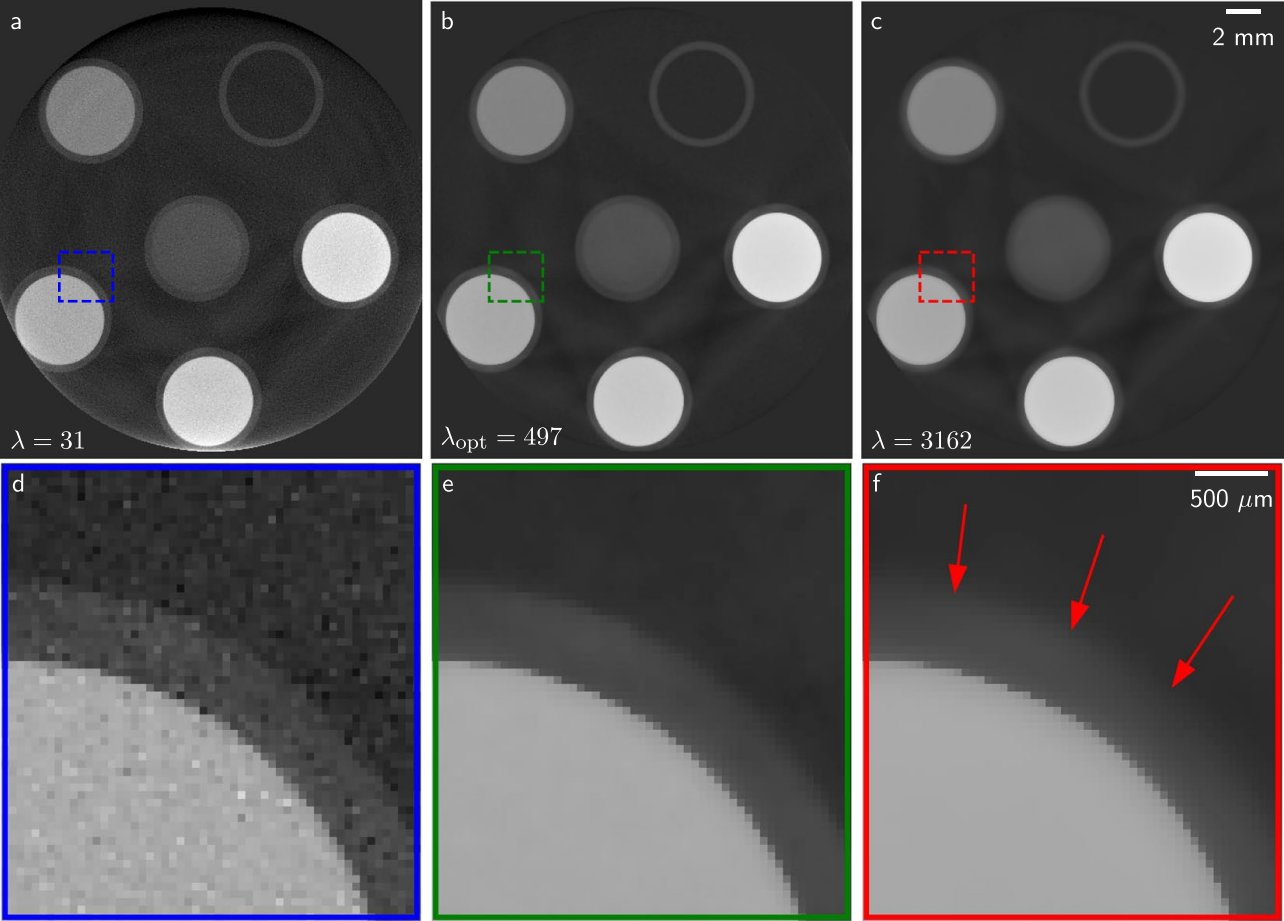
The reconstruction results of the experimental validation. (**a**–**c**) The optimised *λ* parameter is shown in comparison to two regularisation strengths from the parameter sweep. The bottom images (**d**–**f**) show a detail feature of the top row. (**a**) Shows an under-regularised reconstruction with a noisy image. The central image (**b**) shows the reconstruction of the optimised parameter. (**c**) Shows that over-regularisation suppresses noise but also corrupts edges in the image (indicated by red arrows).

The parameter optimisation confirms the findings of the numerical simulation. The optimisation approaches a parameter within the minimum of the histogram entropy provided by the parameter sweep. The changes of regularisation parameter correspond to doubled/halved values with respect to the previous step. This factor corresponds to the largest parameter change allowed by Eqs (6) and (7). During the optimisation procedure, the algorithm reduces the parameter step size. The optimisation starting from ${\lambda }_{{\rm{init}}}=1$ takes the most steps as its initial value is very far away from the final parameter. For very large and strongly over-regularised initial values, the parameter determination fails as it increases further and further (optimisation runs starting from ${\lambda }_{{\rm{start}}}={10}^{5}$). This strong over-regularisation forces the reconstruction to suppress sample features. The resulting image is favoured due to the nature of the histogram entropy which is optimal for histogram delta peaks. However, this issue does not appear when starting from small parameters as the histogram entropy is very sensitive to noise and increases rapidly with increasing noise until uniform value distributions. This ensures the convergence of the algorithm as one can always start from under-regularisation despite the histogram entropy having a global minimum for infinitely large regularisation strengths. The corresponding image result after parameter optimisation is presented in Fig. 7.

Figure 7(a and c) show an under– and over-regularised reconstruction from the parameter sweep. The central Fig. 7(b) shows the final iterative reconstruction after parameter determination. The optimal parameter for histogram entropy is ${\lambda }_{{\rm{opt}}}=497$. The weaker regularisation shows high-frequency noise. The higher regularisation strength shows an over-smoothed image with the edges between the tubes and filling liquids partly vanishing. The central reconstruction shows a balance between noise reduction and feature preservation. In the Supplementary Material the final reconstruction is compared to FBP images obtained using a smooth Hamming filter and a sharp Ramlak filter.

## Summary and Discussion

In this work, we have shown that the regularisation parameter for iterative reconstruction could be determined by optimising an image quality metric such as histogram entropy. The entropy is evaluated close to image edges to be independent of the investigated sample.

The detailed studies for the FORBILD phantom show that the parameter optimisations starting from different initial parameters across the full investigated range end up within the determined histogram entropy minimum. For the numerical data, it was verified that the histogram entropy measure is close to the optimum of reference-based image metrics (RMSE, SSIM). Also for the experimental verification, the parameter optimisation finds the entropy minimum. The optimised regularisation parameters are ${\lambda }_{{\rm{sim}}}=59$ and ${\lambda }_{\exp }=497$ for this simulation study and this experimental measurement, respectively. Their difference can be explained by the differently scaled reconstruction problems (number of detector pixels, number of projection angles, noise in the projections, etc.). However, the algorithm finds the parameter resulting in a similar image impression in both reconstructions without the need for the normalisation of the problem. However, for extreme cases of over-regularised initial parameters, the histogram entropy decreases again letting the determination diverge. This would also be the case for the FORBILD phantom investigations if we used even higher regularisation values as a starting point of the optimisation. In addition, individual samples can have additional local entropy minima because smoothing over small features would create larger features with reduced entropy. Therefore, the parameter determination should always start from under-regularisation to avoid divergence and possibly ‘unwanted’ minima in the over-smoothing regime.

The histogram entropy favours a relatively smooth image. Therefore, the application of the data should fit a smooth image, e.g., use for post-segmentation or as a basis for rendering surfaces, which is normally complicated by image noise. For diagnostic use in medical imaging, the entropy-guided approach probably favours overly smooth reconstructions. However, for diagnostic and other uses other image metrics could be investigated, leaving the tuning concept the same. In the event of poor projection quality with a lot of noise, this would also translate into very noisy initial and FBP reconstructions. However, the entropy approach does not account for this, so the favoured reconstruction would be as smooth as the images in this example. This could lead to possible feature loss or feature creation from the image noise yet the resulting image remains very smooth, hiding how ill-posed the reconstruction problem is.

Based on these observations, this parameter optimisation method can also be tested with other image metrics behaving differently for different noise levels or favouring other smoothness regimes. For example, a metric based on noise and edge sharpness properties could be tuned to favour different smoothness levels. Additionally, this approach could be tested to tune the strength as well as the turning point *γ* of the Huber regularisation in an alternating fashion. Further improvements to the algorithm could include an evaluation of the optimum stabilisation period. A more advanced means of selecting weaker and stronger regularisation parameters could be investigated. This could be realised by fitting a quadratic potential to the histogram entropy values for the three parallel reconstructions to refine parameters for subsequent optimisation steps instead of bisecting the interval. The combination with solvers other than the OGM could also improve performance as the stabilisation process might behave completely differently for solvers not applying a curvature.

In summery, the proposed parameter optimisation method determines reliably and robustly the regularisation strength resulting from an optimum image quality metric.

## Supplementary information

Supplementary Material: Metric-guided regularisation parameter selection for statistical iterative reconstruction in computed tomography

## Data Availability

The experimental data is available from the corresponding author Sebastian Allner (S.A.) on request.
