# Supplementary material for: Metric-guided regularisation parameter selection for statistical iterative reconstruction in computed tomography
Source: Sci Rep. 2019 Apr 12;9:6016. doi: 10.1038/s41598-019-40837-7 (PMC6461679; doi:10.1038/s41598-019-40837-7)
Supplement: Supplementary file 1 — Supplementary Material: Metric-guided regularisation parameter selection for statistical iterative reconstruction in computed tomography [file 41598_2019_40837_MOESM1_ESM.pdf]

Supplementary Material: Metric-guided regularisation parameter selection for statistical iterative reconstruction in computed tomography

Sebastian Allner[1,*], Alex Gustschin[1], Andreas Fehringer[2], Peter B. Noël[3], and Franz Pfeiffer[1, 3]

[1]Chair of Biomedical Physics and Department of Physics and Munich School of BioEngineering, Technical University of Munich, 85748 Garching, Germany
[2]MITOS GmbH, 85748 Garching, Germany
[3]Department of Diagnostic and Interventional Radiology, Klinikum rechts der Isar, Technical University of Munich, 81675 München, Germany
[*]Sebastian.Allner@tum.de

1 Comparison to Analytical Reconstructions

In the main manuscript, we display iterative reconstruction results calculated with different regularisation parameters. In this supplementary material, the performance of the 'best' image with the determined parameter is compared to conventional analytical reconstructions. Fig. 1 displays the reconstructions for the numerical simulation.

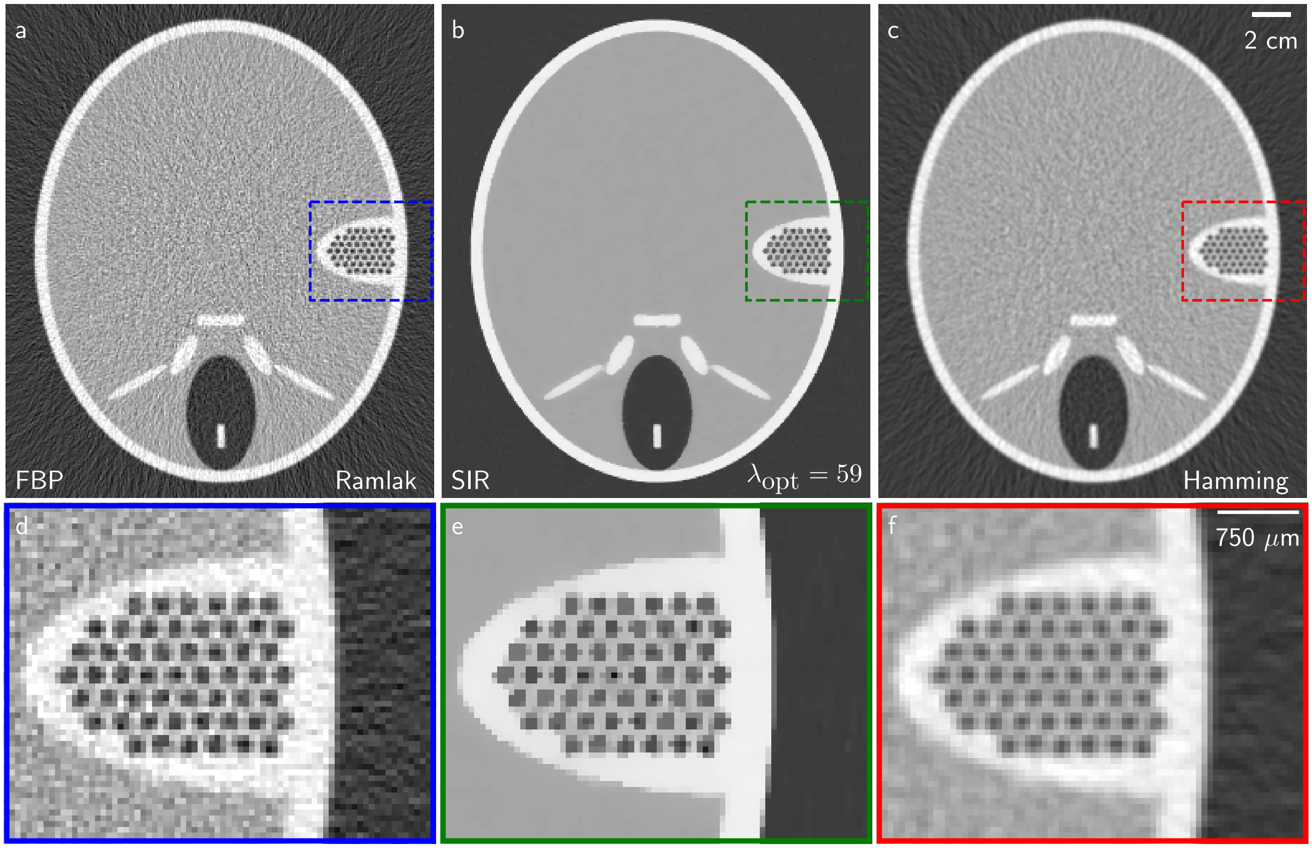

Figure 1. Comparing reconstruction results of the numerical FORBILD phantom study. a) FBP with Ramlak filter kernel, b) the final reconstruction with the determined parameter, and c) FBP with Hamming filter kernel.

The FBP is calculated with the Ramlak filter, a sharp filter kernel without noise suppression, and with the Hamming filter kernel, which has smoother image appearance. The Ramlak-FBP entails a large amount of noise that is suppressed in the Hamming-FBP by reducing the influence of high-frequency components. However, this affects also the edges of the image and the Hamming-FBP has a blurred image appearance. The iterative result features sharp edges as well as suppressed noise.

A corresponding comparison is conducted in Fig. 2.

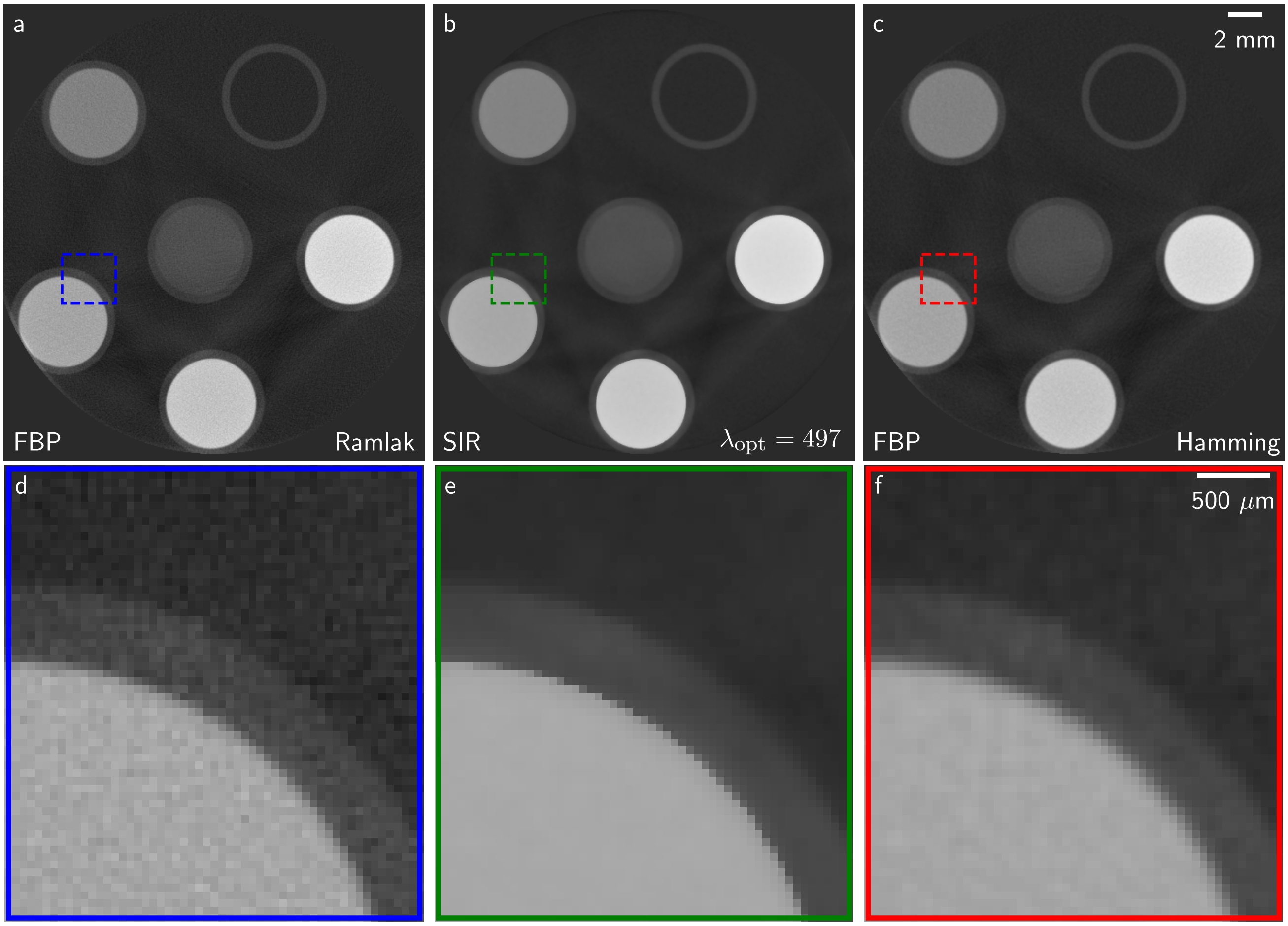

Figure 2. Comparing reconstruction results of the experimental measurement. a) FBP with Ramlak filter kernel, b) the final reconstruction with the determined parameter, and c) FBP with Hamming filter kernel.

The outcome of the experimental measurement data supports the same conclusion as for the numerical study. The Ramlak-FBP has a sharp image appearance but a high noise amplitude. The Hamming-FBP suppresses noise but also smooths edges. The iterative result with the regularisation parameter determined with the method introduced in the main manuscript features good edge preservation and noise suppression.

2 Image stabilisation after changing regularisation strength

After changing a regularisation parameter it takes a number of iterations before the reconstruction adapts to the change. This is because the data and regularisation terms having fundamentally different curvatures. The regulariser has a constant and the data term a hyperbolic curvature. This causes the iterative reconstruction to converge at different velocities for different regions of the image. Therefore, a stabilisation period is needed for this combination of iterative reconstruction and solver. Other solvers such as NLCG may behave differently. In this work the entropy is tracked during the stabilisation period of 35 iterations (also called optimisation step). One example for such an optimisation step is given in figure 3.

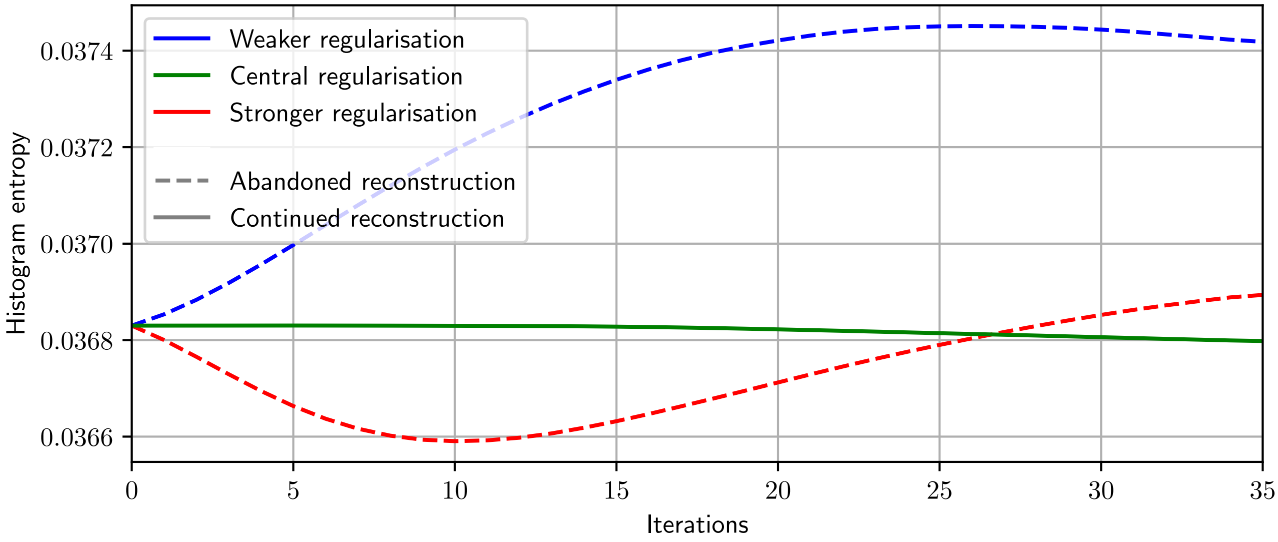

Figure 3. The image entropy of an exemplary optimisation step of 35 iterations to stabilise the reconstruction after changing the regularisation parameter. This plot shows the fifth optimisation round for $\lambda_{init} = 1$. The central regularisation parameter produced the 'best' entropy and is retained. For the next step, the surrounding parameters are selected with closer values.
